# Commentary: Association between pretransfer cleavage-stage blastomere dynamics and pregnancy outcomes in fresh single embryo transfer cycles: a retrospective cohort study

**DOI:** 10.3389/fendo.2026.1720455

**Published:** 2026-02-05

**Authors:** Bangbei Wan, Weiying Lu

**Affiliations:** 1 Reproductive Medical Center, Hainan Women and Children’s Medical Center, Haikou, China; 2 Department of Urology, Haikou Affiliated Hospital of Central South University Xiangya School of Medicine, Haikou, China

**Keywords:** embryo, mammalian, blastomeres, embryo transfer, fertilization *in vitro*, pregnancy outcome

## Introduction

Wang et al. (1) examined whether (i) blastomere count immediately before transfer and (ii) short-interval cleavage on the morning of transfer (07:00–11:00) predict outcomes in fresh single embryo transfer (SET). In 561 cycles, clinical pregnancy and live birth increased with higher pre-transfer cell numbers; notably, 9–10-cell embryos that cleaved again between 07:00 and 11:00 outperformed those without interval increase. Multivariable models confirmed lower odds for ≤7-cell embryos vs 8-cell, and identified endometrial thickness as an independent correlate of success. These results speak to a practical day-3 decision point: can a brief, standardized re-check just before transfer refine selection beyond static morphology?

## Study design and interpretability

### Strengths

The focus on fresh SET limits multi-embryo confounding, and adding a second observation window (≈4 h later) is clinically intuitive and low-cost. Reporting adjusted odds ratios and including endometrial parameters enhances bedside interpretability (1).

### Context

An expanding literature suggests that greater day-3 blastomere number tracks with better outcomes in fresh day-3 transfers, challenging the notion that faster cleavage is uniformly detrimental (2–4). Time-lapse work also links orderly, ongoing divisions to implantation potential, supporting dynamic (not merely static) assessment (5, 6). Still, evidence is mixed historically—some earlier cohorts found day-3 cell number alone poorly discriminative—so combining cell number with qualitative morphology remains prudent (7).

### Limitations

As a single-center retrospective analysis, confounding by indication is plausible (e.g., embryologists might preferentially schedule/choose embryos based on morning features). Prospective protocols that pre-specify 07:00→11:00 triage rules and blind embryologists to interim dynamics could mitigate bias (1).

### Measurement window, embryo physiology, and potential trade-offs

Wang et al. (1) show that short-interval cleavage (07:00→11:00) in 9–10-cell embryos associates with higher clinical pregnancy and live birth, consistent with the premise that continuing, non-erratic divisions reflect robust metabolism and genome activation (5, 6). Conversely, literature cautions that aberrantly rapid/atypical cleavage (e.g., direct 1→3) can signal chromosomal error; such embryos are best cultured onward rather than selected for immediate transfer (8, 9). Therefore, interval gain should be interpreted alongside fragmentation, symmetry, and multinucleation, forming a composite “green-light” rather than a single-feature rule.

Two practical clarifications would aid reproducibility: (i) exact time stamps from insemination/ICSI to both assessments and to transfer (reducing clock variability), and (ii) rules for adjudicating borderline increases (distinguishing true cytokinesis from observation artifacts) (1).

Independent data show that briefly extending day-3 culture by ~7–8 h (a window comparable to 07:00→11:00) can improve selection efficiency and outcomes—supporting the biological plausibility that near-transfer dynamics carry actionable signal (10).

## Clinical implications and workflow

### Triage when multiple embryos qualify

The findings support prioritizing embryos that are ≥8-cell and demonstrate interval cleavage just before transfer—especially within the 9–10-cell subset where the effect was most pronounced (2–4). Such a “dynamic gain” tiebreaker is simple to implement without new equipment and aligns with time-lapse insights (5, 6).

### Counseling

The ≤7-cell group’s inferior outcomes warrant transparent counseling about reduced implantation prospects and consideration of deferring transfer if feasible (2–4). Reassuringly, among live births in Wang et al., neonatal metrics did not differ by cell-count strata (1).

### Endometrium matters

Given the study’s independent association of endometrial thickness with success, documenting EMT at the morning check is sensible; contemporary reviews and meta-analyses indicate that EMT relates to outcomes in a non-linear fashion and that reporting thresholds/curves improves comparability across centers (11, 12).

### Standardization

To generalize adoption, centers should standardize: timing of morning checks, double-observer grading, handling conditions between assessments, and documentation of EMT. Where available, time-lapse can validate whether manual 07:00→11:00 increases represent genuine cleavage sequences (5, 6).

## Discussion

Wang et al. provide actionable evidence that pre-transfer blastomere count and very short-term cleavage dynamics add clinically useful signal in fresh day-3 SET (1). Practically, if two embryos look similar at 07:00, preference for the one that continues to cleave by 11:00—particularly when starting at 9–10 cells—is reasonable and low-overhead. The work complements consensus that 8-cell is a helpful benchmark while emphasizing that “8 is not a ceiling” when dynamic gain is present. Future prospective, protocol-level studies should test morning-of-transfer dynamic assessment vs static morphology alone, incorporate full morphology and endometrial parameters, and explore integration with time-lapse trajectories.
